# Evidence for a pre-malignant cell line in a skin biopsy from a patient with Nijmegen breakage syndrome

**DOI:** 10.1186/s13039-018-0364-6

**Published:** 2018-02-07

**Authors:** Raneem Habib, Heidemarie Neitzel, Aurelie Ernst, John K. L. Wong, Bozenna Goryluk-Kozakiewicz, Antje Gerlach, Ilja Demuth, Karl Sperling, Krystyna Chrzanowska

**Affiliations:** 1 Department of Human Genetics, Ruhr-University Bochum, Bochum, Germany; 2 Institute of Medical and Human Genetics, Charité - Universitaetsmedizin Berlin, Berlin, Germany; 3 Division of Molecular Genetics, German Cancer Research Center (DKFZ), Heidelberg, Germany; 4 Department of Medical Genetics, The Children’s Memorial Health Institute, Warsaw, Poland; 5 Lipid Clinic at the Interdisciplinary Metabolism Center, Charité - Universitaetsmedizin Berlin, Berlin, Germany

**Keywords:** Nijmegen breakage syndrome, Cell line, Unbalanced translocation, Proliferative advantage

## Abstract

**Background:**

Nijmegen breakage syndrome is an autosomal recessive disorder characterized by microcephaly, immunodeficiency, hypersensitivity to X-irradiation, and a high predisposition to cancer. Nibrin, the product of the *NBN* gene, is part of the MRE11/RAD50 (MRN) complex that is involved in the repair of DNA double strand breaks (DSBs), and plays a critical role in the processing of DSBs in immune gene rearrangements, telomere maintenance, and meiotic recombination. NBS skin fibroblasts grow slowly in culture and enter early into senescence.

**Case presentation:**

Here we present an incidental finding. Skin fibroblasts, derived from a 9 year old NBS patient, showed a mosaic of normal diploid cells (46,XY) and those with a complex, unbalanced translocation. The aberrant karyotype was analysed by G-banding, comparative genomic hybridization, and whole chromosome painting. The exact breakpoints of the derivative chromosome were mapped by whole genome sequencing: 45,XY,der(6)(6pter → 6q11.1::13q11 → 13q21.33::20q11.22 → 20qter),-13. The deleted region of chromosomes 6 harbors almost 1.400 and that of chromosome 13 more than 500 genes, the duplicated region of chromosome 20 contains about 700 genes. Such unbalanced translocations are regularly incompatible with cellular survival, except in malignant cells. The aberrant cells, however, showed a high proliferation potential and could even be clonally expanded. Telomere length was significantly reduced, *hTERT* was not expressed. The cells underwent about 50 population doublings until they entered into senescence. The chromosomal preparation performed shortly before senescence showed telomere fusions, premature centromere divisions, endoreduplications and tetraploid cells, isochromatid breaks and a variety of marker chromosomes. Inspection of the site of skin biopsy 18 years later, presented no evidence for abnormal growth.

**Conclusions:**

The aberrant cells had a significant selective advantage in vitro. It is therefore tempting to speculate that this highly unbalanced translocation could be a primary driver of cancer cell growth.

**Electronic supplementary material:**

The online version of this article (10.1186/s13039-018-0364-6) contains supplementary material, which is available to authorized users.

## Background

Nijmegen breakage syndrome (NBS) is an autosomal recessive disorder characterized by chromosome instability associated with microcephaly, immunodeficiency, hypersensitivity to X-irradiation, and a high predisposition to cancer. The majority of NBS patients are of Central and Eastern European origin and carry the common founder mutation in the *NBN (NBS1)* gene, c.657_661del5 [1–3]. By the age of 20 years more than 40% of patients have developed a malignant disease, predominantly of lymphoid origin [4–6]. Even heterozygous carriers of the founder mutation have an increased cancer risk [7, 8]. Nibrin, the product of the *NBN* gene, is part of the MRE11/RAD50 (MRN) complex that is involved in the repair of DNA double strand breaks (DSBs), both by homologous recombination repair (HRR) and non-homologous end-joining (NHEJ). Moreover, the *NBN* gene plays a critical role in the processing of DSBs in immune gene rearrangements, telomere maintenance, and meiotic recombination [9–11]. Thus, almost all aspects of the DNA damage response (DDR), including apoptosis, are affected [12].

NBS skin fibroblasts grow slowly in culture and enter early into senescence. Consequently, they have a low reprogramming efficiency into induced pluripotent stem cells (iPSCs) [13]. The iPSCs show, amongst others, numerous chromosomal aberrations, a delayed response to DSB induction, slower growth rate, and a reduced apoptotic response to stress [14].

Here we present a, perhaps unique, incidental finding. Skin fibroblasts, derived from a 9 years old NBS patient showed a mosaic of normal diploid cells and those with a complex, unbalanced translocation, leading to the loss and duplication of hundreds of genes. Such primary events are regularly incompatible with cellular survival. Unexpectedly, these cells showed a high proliferation potential in vitro and could even be cloned. Thus, this complex aberration might represent an early (first) step toward malignant transformation.

## Case presentation

The skin biopsy was obtained from a 9 years old Polish child with NBS. He presented all phenotypic characteristics of NBS, i.e. microcephaly and typical facial features, recurring pulmonary infections, bronchiectasis, and combined humoral and cellular immunodeficiency. The cell line was established with the ethical approval of The Children’s Memorial Health Institute, Warsaw. At the age of 21 years the patient underwent total thyroidectomy (papillary thyroid carcinoma, follicular variant). The patient was under systematic longitudinal observation and treatment at The Children’s Memorial Health Institute from the age of 9 until 21 years. The patient was contacted again at the age of 27 years. There was only a scar at the site of the skin biopsy without any evidence of abnormal growth.

## Results

**Fig. 1:**
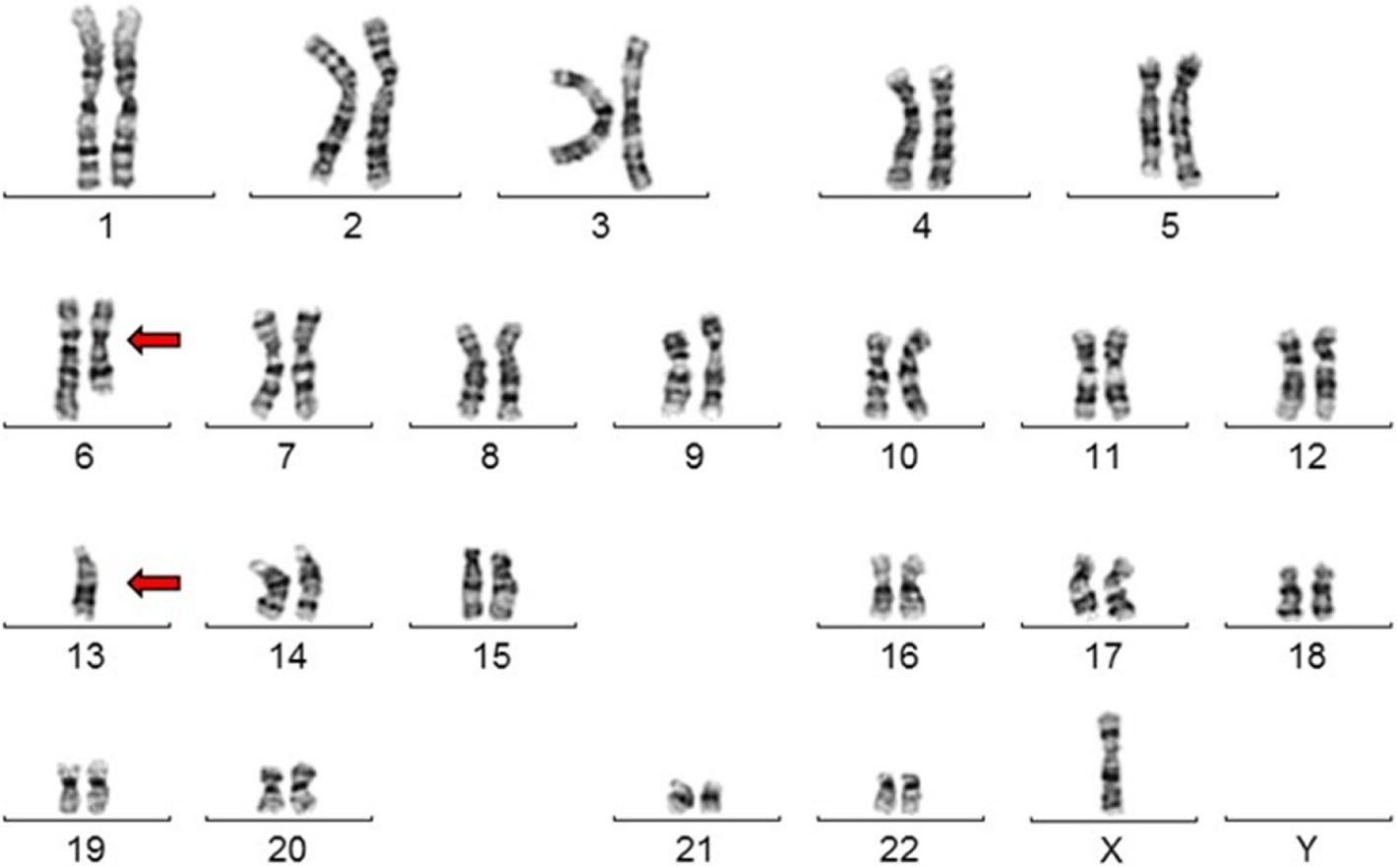
Karyotype of the aberrant NBS line 94P0496 after G-banding. The arrows point to the derivate chromosome and the one chromosome 13. The chromosomal preparation was performed after cloning, i.e. after about 40 cell divisions. The Y chromosome has been lost during cultivation

The fibroblast cell line (94P0496) was derived from the NBS patient, homozygous for the common founder mutation in the *NBN* gene, a five-base-pair deletion, c.657_661del5 in exon 6, that leads to two truncated protein fragments, p26- and p70 Nibrin. After chromosome analysis two cell lines could be identified, one with a normal diploid karyotype, 46,XY, the other with 45 chromosomes. The latter had only one chromosome 6 and 13 plus a derivative chromosome (Fig. 1).

**Fig. 2:**
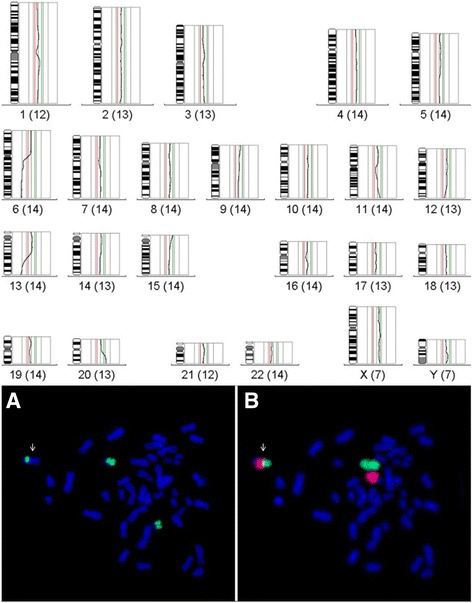
Characterization of the aberrant line 94P0496 after comparative genomic hybridization and whole chromosome painting. Above: CGH analysis of the aberrant cell line 94P0496 showing partial monosomy for the q arms of chromosome 6 and 13 and partial trisomy for chromosome 20. Below: Metaphase with marker chromosome (white arrow) after whole chromosome painting. A: chromosome 20 in green; B: chromosomes 13 in red and 6 in green (Original from [16])

As shown by whole chromosome painting (WCP) the p-arm including the centromere of the derivative chromosome is from chromosome 6, the proximal part of the long arm from chromosome 13 to which the q-arm of chromosome 20 is attached, resulting in partial monosomy of chromosome 6 and 13 and partial trisomy of chromosome 20 (Fig. 2). This was confirmed by comparative genomic hybridization (CGH, Fig. 2).

**Fig. 3:**
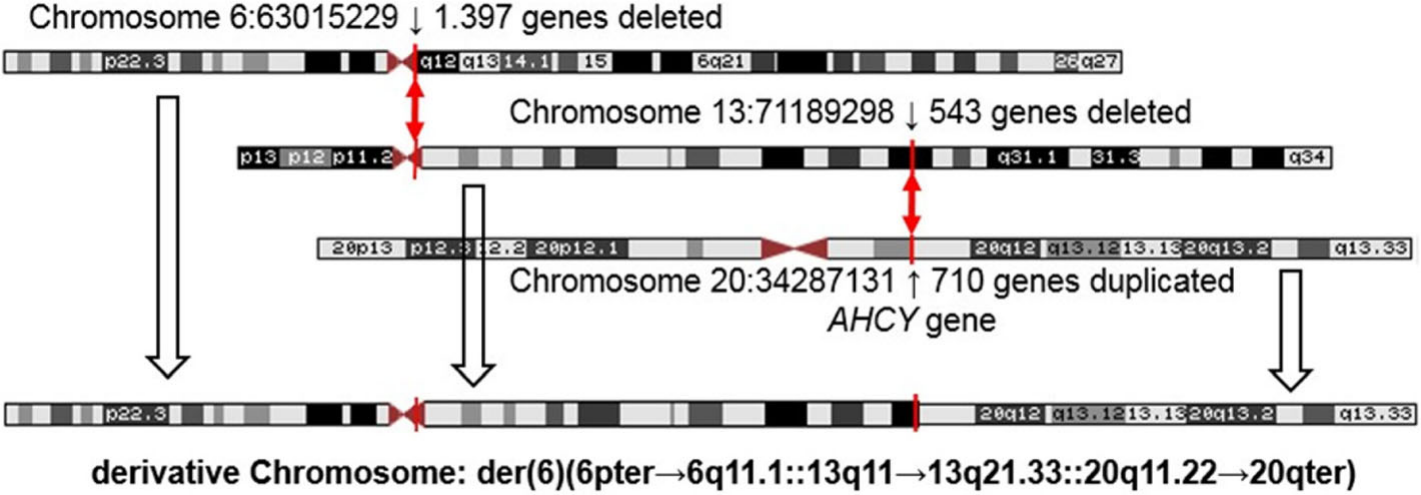
Reconstruction of the derivative chromosome after whole genome sequencing. The exact breakpoints are depicted and the number of deleted and duplicated genes indicated (NCBI, Map Viewer, Annotation Release 108)

The exact breakpoints were mapped by whole genome sequencing (WGS; Additional file 1: Figure S1a,b). The breakpoint on the derivative chromosome 6 is on 6q at chr6:63,015,229. Attached to this breakpoint is a chromosome 13-specific satellite I DNA, mapped to chromosome 13 in 13p13 and the pericentromeric region [15]. The distal breakpoint on chromosome 13 is at chr13:71,189,298 followed by the translocated segment of the q-arm of chromosome 20 starting at position chr20:34,287,131 to 20qter. Based on FISH analysis with the XL acro-p probe and G-banding there was no evidence for an interstitial short arm of chromosome 13. Thus, we assume that the break was in the subcentromeric band at 13q1.1. The karyotype of this complex unbalanced translocation according to ISCN is: 45,XY,der(6)(6pter → 6q11.1::13q11 → 13q21.33::20q11.22 → 20qter),-13. The deleted region of chromosome 6 harbors 1.397 genes, the deleted region of chromosome 13 contains 543 genes, and the duplicated region of chromosome 20 encompasses 710 genes (Fig. 3). Such unbalanced translocations are regularly incompatible with cellular survival, except in malignant cells.

The cell cycle length was analysed after BrdU labelling for 36 and 72 h. Based on the labelling pattern of the sister chromatids (Additional file 1: Figure S2), the diploid and aberrant cells were classified if they had passed one, two or three S-phases after labelling (Table 1). The number of M2 cells after 36 h and of M3 cells after 72 h was significantly higher for the aberrant cells and consequently their cell cycle was shorter than that of the diploid cells (*P* < 0.05).

**Table 1:** Analysis of metaphases of the 94P0496 cell line after 36 and 72 h of BrdU labelling

BrdU incubation	Mitoses after BrdU labelling	Metaphases analysed	aberrant karyotype	normal karyotype
36 h	M1	5	3	2
	M2	50	48	2
72 h	M1	0	0	0
	M2	16	13	3
	M3	34	34	0

The cells were classified if they had passed one (M1), two (M2) or three S-phases (M3) after labelling. The aberrant cells underwent significantly more cell divisions than those with the normal karyotype (Fisher’s exact test, *P* < 0.05; Original from [16]).

At an early passage the diploid and aberrant cells were analysed for chromosomal aberrations after irradiation in G2-phase with 0 Gy, 0.5 Gy and 1.0 Gy. About 20 normal and 40 aberrant cells were analysed each. The types of aberrations were classified as achromatic lesions, chromatid and isochromatid breaks, and chromatid translocation (Additional file 1: Figure S3). In order to calculate the total number of chromatid breaks per cell the latter were counted twice, the achromatic lesions neglected. The number of chromosomal aberrations was significantly higher in the aberrant cells than in the normal ones after irradiation (*P* < 0.05; Table 2). Thus, chromosomal instability of the aberrant line was not decreased but increased.

**Table 2:** Analysis of chromosomal breaks in normal and aberrant cells of the 94P0496 cell line after irradiation

Dose	94P0496 cells	Metaphases analysed	Chromatid breaks	Chromatid translocat.	Chromos. breaks	Total Breaks	Breaks/ metaphase
0 Gy	normal	23	5	–	–	5	0.22
	aberrant	42	10	–	2	12	0.29
0.5 Gy	normal	20	23	–	6	29	1.45
	aberrant	42	53	4	9	66	1.57
1.0 Gy	normal	21	39	–	10	49	2.3
	aberrant	43	125	6	13	144	3.4

The aberrant cells showed more aberrations than those with the normal diploid karyotype (Chi-square test, *P* < 0.05; Original from [16]).

**Fig. 4:**
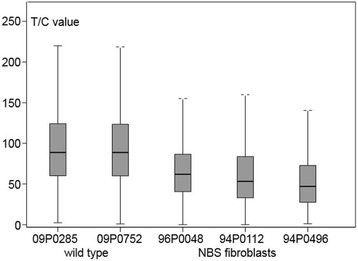
Boxplots of telomere lengths of normal diploid and NBS-fibroblasts. The telomere length (T/C value) of the three NBS cell lines, incl. 94P0496, is significantly shorter than that of the two normal fibroblast cell lines, tested at passage 7 (Mann-Whitney test; *P* < 0.05; Original from [16])

Moreover, telomere length (T/C value) was measured by Q-FISH in 94P0496 and two other NBS fibroblast lines in comparison with 2 control fibroblast cell lines at passage 7. The telomere length was significantly longer in the controls than in the NBS-fibroblasts (*P* < 0.05) with median values (T/C values) of 87.9 and 87.5 for the controls and 48.5 for 94P0496 (Fig. 4). Thus, the T/C value of 94P0496 was reduced to about 55%. In addition, the absolute telomere length was measured by Terminal Restriction Fragment (TRF) analysis. This technique is based on Southern blots and also included the subtelomeric region. The NBS fibroblasts had a telomere length of about 12.5 kb compared to about 17 kb for the control fibroblast [16]. There was no detectable *hTERT* expression in the NBS cell line 94P0496 in contrast to two SV40 transformed NBS cell lines (Additional file 1: Figure S4).

The cells were cultivated until they reached senescence in two independent culture attempts. Regularly some subcultures were kept confluent for several weeks to test if some cells escaped contact inhibition. This, however, was not the case. In the second attempt, the cells were cloned and fourteen clones propagated. Thirteen clones were diploid, one tetraploid, all contained the derivative chromosome 6 and one normal chromosome 13 (Fig. 1). After confluency the cultures were split at a 1:4 ratio until the cells entered into senescence six to ten passages later. Altogether we calculate that the cells underwent about 50 cell divisions until they reached senescence, i.e. at least ten division after establishment of the cell line from tissue pieces until the cells could be propagated for the first time. After four passages they were cloned. Thereafter, the cells underwent at least 20 cell divisions until the flasks were confluent. After further 12 to 20 cell divisions the cells entered into senescence.

**Fig. 5:**
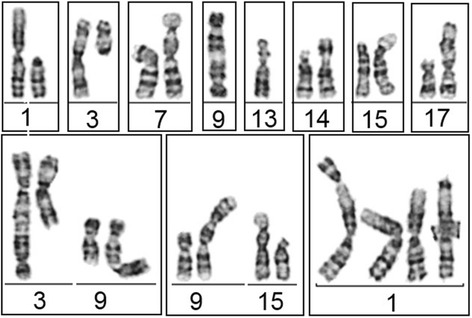
Pattern of chromosomal aberrations, especially telomere fusions, observed in the aberrant clones during log. growth

**Fig. 6:**
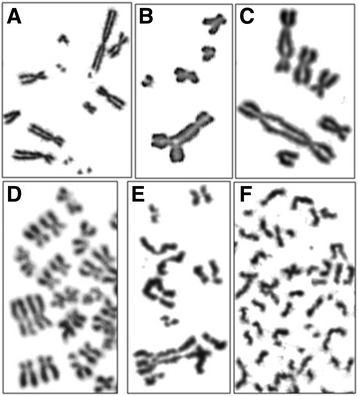
Pattern of chromosomal aberrations observed in the aberrant clones immediately before entering into senescence. A: multiple isochromatid breaks; B: triradial; C: two dicentric chromosomes (telomere fusion); D: endoreduplication; E and F: premature centromere division

The first chromosomal preparation was performed when the cells were still in logarithmic growth and three G-banded metaphases were analysed each. Only three clones showed in at least two metaphases the original karyotype. The others had additional aberrations, such as telomere fusions and marker chromosomes (Fig. 5). In seven of the 42 metaphases the Y chromosome has been lost. Shortly before the cells entered into senescence the second chromosome preparation was performed. Only in eleven of the fourteen clones some metaphases could be found. All had complex aberrations, such as premature centromere divisions, endoreduplications, tetraploidizations, isochromatid breaks, and a variety of marker chromosomes (Fig. 6).

**Fig. 7:**
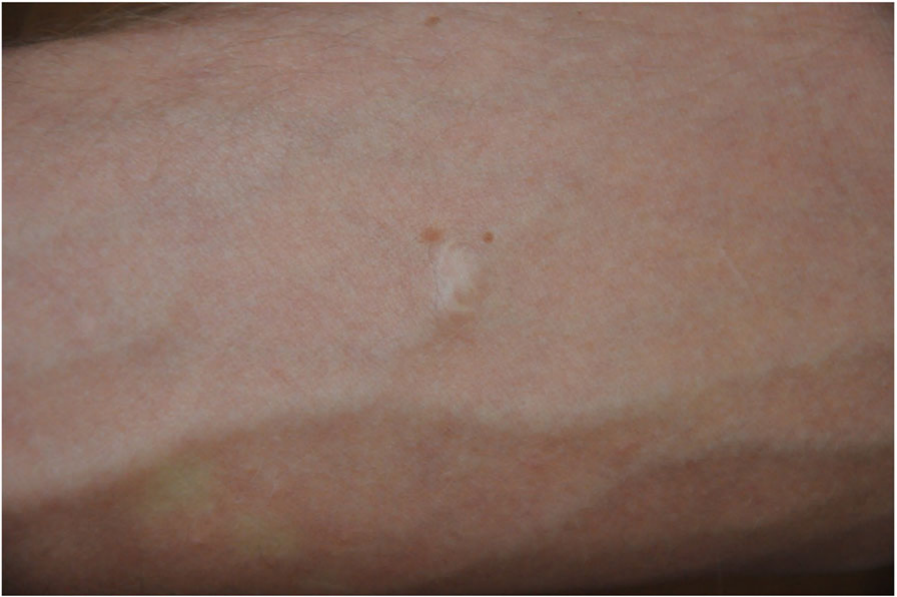
Scar after skin biopsy 18 years before. There is no evidence for abnormal growth

The examination of the patient 18 years later showed no abnormal growth at the site of the skin biopsy (Fig. 7).

## Discussion

The MRN complex is central to the maintenance of genomic stability by the DNA damage response (DDR) network, which is, amongst others, involved in cell cycle checkpoint control, telomere maintenance, and apoptosis. Thus, NBS patients with defective DDR show a highly increased cancer risk due to their massive genetic/chromosomal instability. This leads to a large number of genetically different cells from which those capable of continual proliferation will be selected. Clearly, human tumor cells often show gross chromosomal abnormalities. The question, however, is whether these changes are primary events or secondary epiphenomena reflecting the genomic instability of these cells [17, 18]. Without any doubt, the growth advantages conveyed by specific chromosomal translocations as in chronic myelogenous leukemia and Burkitt’s lymphoma leading to oncogenic fusion proteins or overexpression of oncogenes are primary drivers of cell growth. The prevailing view, however, is that most of the complex chromosomal changes of malignant cells are secondary events. In this context the aberrant NBS fibroblast cell line is of special interest. The percentage of these cells increased during cultivation and they replaced the diploid cells, which is, at least partially, explained by its shorter cell cycle. Clearly, this line had a selective advantage in tissue culture, even despite its increased chromosomal instability. Moreover, these cells could be cloned. This is in contrast to NBS skin fibroblasts, which grow slowly in culture, enter early into senescence and have an extremely low reprogramming efficiency [13]. There are strong arguments for the assumption that the derivative chromosome is the crucial, primary event for the selective advantage of this cell line against the NBS background. It was the only cytogenetic aberration detected immediately after establishment of the cell culture. At the third passage after establishment about 50% of the cells showed the aberrant karyotype, which continuously took over. The derivative chromosome was also present in all 14 clones until senescence.

This complex chromosomal translocation results from the rearrangements between three chromosomes. Such events depend on DNA double-strand breaks and misrepair of the broken chromatin ends [19]. The major DNA repair pathways are Homologous Recombination (HR) and Non-homologous End-Joining (NHEJ) [20]. HR utilizes sister chromatids as a template and is error proof, while the NHEJ process directly ligates the broken DNA ends and is error prone. The flanking sequences of the translocation breakpoints did not show any alterations (see Additional file 1: Figure S1a, b) pointing to HR as possible underlying mechanism.

The telomere length was reduced in the aberrant cell line which is a characteristic of NBS cells in general [21]. Quite surprisingly these cells underwent about 50 cell divisions until they entered into senescence. Telomerase was not expressed, however, we cannot exclude that a telomere maintenance pathway, such as alternative lengthening of telomeres (ALT), was at least temporarily activated. Clearly, the telomeres became shorter during cultivation. The shortened telomere ends are signaled as DSBs leading to telomere fusions and chromosomes with two centromeres in the NBS cells. This results in breakage-fusion-bridge cycles, because the different centromeres may be pulled in opposite directions during anaphase [20]. Thus, the cells acquired numerous additional, non-recurrent chromosomal aberrations apart from those that are due to the inherent chromosomal instability. Interestingly, this took place already during the phase of logarithmic cell growth (see Fig. 5). In contrast to normal diploid cells, in the aberrant cells the DDR did not trigger the pathway to apoptosis. The impairment of apoptosis and cell cycle checkpoint control is a hallmark of NBS cells in general and was also observed in induced pluripotent stem cells after reprogramming NBS fibroblasts [14]. It could be at least one explanation for the late entry into senescence. However, faced with the enormous imbalance generated by the origin of the derivative chromosome, the observed massive growth advantage is still an open question.

Almost 2.000 genes were lost and 710 genes from chromosome 20 gained. Gain of chromosome 20 is frequently observed in colorectal carcinomas and malignant epithelial tumors [22, 23]. Moreover, the combination of gains of 20q and losses of 6q and 13q is common in oral squamous cell carcinomas [24]. The duplicated 20q region contains the putative oncogenes *AURKA* and *ZNF217*, as well as the transcription factor E2F1 that is involved in DSB repair in association with the MRN complex [25]. Loss of the *IGF2R* on 6q increases the growth of human and murine tumors [26]. It should be noted that loss of chromosome 14 confers a growth advantage to NBS-iPSCs [14]. These few examples, however, only illustrate how gains and losses of specific genes/chromosomes could influence cell proliferation and malignant transformation. In this context it is of interest that a high frequency of spontaneous, nonspecific, and even complex translocations were found in lymphocytes and lymphoblastoid cell lines from NBS patients. Moreover, exactly as in our case two clones with such a translocation showed an increased rate of proliferation and the authors pointed out that such clones could serve as an in vitro model for tumorigenesis [27].

## Conclusions

(Partial) aneuploidy of single chromosomes is detrimental to diploid cells, but could be “beneficial” in cancer cells. Most solid cancer show large-scale chromosomal alterations and it is generally accepted that some are functionally important (cancer drivers) whilst others represent only random changes. Based on our findings we come to the conclusion that the derivative chromosome might be a driver aberration and represent an early (first) step in malignant transformation against the NBS background of chromosomal instability. This premise, however, can be tested empirically by reconstitution of telomerase activity in these cells and study if this combination of the derivative chromosome and telomerase confers a tumorigenic phenotype, just in line with the statement of Bunting and Nussenzweig [17] “Making sense of how translocations influence cancer cell growth will be a major topic of research interest in the coming years.”

## Methods

### Cytogenetics

The fibroblast were grown and cloned in Amniomax medium at 37 °C and 5% CO_2_, supplemented with the usual amount of antibiotics.

G-banded metaphases were prepared according to standard clinical laboratory procedures. Comparative genomic hybridization (CGH) and whole chromosome painting (WCP) was performed according to Tönnies et al. [28]. Fluorescence in situ hybridization (FISH) was performed with the XL acro-p probe, which specifically hybridize to the p-arms of all human acrocentric chromosomes, according to manufacturer’s instruction (MetaSystems). The length of telomeres was measured by quantitative fluorescence in situ hybridization of telomere repeats (Q-FISH) according to Perner et al. [29]. It is based on the fluorescence intensity of single telomeres (T) relative to a constant repetitive region in the centromeric region of chromosome 2 (C). The T/C ratio reflects the relative length of individual telomeres. In addition, the total relative length of all chromosomes was estimated.

Based on Bromodeoxyuridine labeling (BrdU) for 36 h and 72 h the number of cells in the first, second and third mitosis was analyzed after Hoechst 33,258 staining using a florescence microscope (Axioscope) and the software of Metasystems. The number of chromosomal aberrations was calculated 4 h after X-irradiation with 0.5 and 1.0 Gy (Muller MG 150 X ray apparatus; U_A_, 100 kV; I, 10 mA; filter, 0.3 mm Ni; dose rate 2.1 Gy/min), including 2 h Colcemid treatment.

### Molecular genetics

The NBN founder mutation was studied by means of exon 6 PCR and sequencing.

Absolute telomere length was measured by Terminal Restriction Fragment (TRF) length analysis, performed according to the standard manufacturer’s instruction of Roche (TeloTAGGG telomere length assay). The protocol involved DNA cutting into fragments by a mixture of frequently cutting restriction enzymes: Hinf1 and Rsa1, separating the fragments by gel electrophoresis, and blotting on a nylon membrane. The blotted DNA fragments were hybridized to a digoxigenin (DIG)-labeled probe specific for telomeric repeats, incubated with a DIG-specific antibody, exposed to an x-ray film to estimate the mean TRF length.

Expression of the human telomerase reverse transcriptase gene (hTERT) was analyzed by qPCR. The hTERT cDNA was synthesized according to the standard manufacture’s instruction (Invitrogen). GAPDH (glyceraldehyde-3-phosphatedehydrogenase) was used as an internal, HPRT (guanine phosphoribosyl transferase) as endogenous control gene. All PCRs were performed on Applied Biosystem prism 7500 (software DSD V1.2.3). The qPCR products were checked by agarose gel electrophoresis, visualized by UV-transilluminator and photographed.

Detailed protocols of the cytogenetic and molecular genetic methods are presented in Habib, 2012 [16].

Whole genome sequencing was performed using the Illumina X Ten platform. Purified DNA was quantified using the Qubit Broad Range double-stranded DNA assay (Life Technologies, Carlsbad, CA, USA). Genomic DNA was sheared using an S2 Ultrasonicator (Covaris, Woburn, MA, USA). Whole- genome sequencing and library preparations were performed according to the manufacturer’s instructions (Illumina, San Diego, CA, USA or NEBNext, NEB). The quality of the libraries was assessed using a Bioanalyzer (Agilent, Stockport, UK).

### Statistical tests

The original data were exported to Excel 2007, GraphPad Prism 5 software and SPSS15.0 software for graphs and boxplots, statistical tests as Mann-Whitney, Fisher’s exact and Chi- square test.

## Additional file

Additional file 1: Figure S1a,b. Alignment of the cDNA spanning the breakpoints at chromosomes 6 and 13 and chromosomes 13 and 20. **Figure S2**. Metaphases of the NBS cell line after BrdU labelling for 36 and 72 h. **Figure S3**. Metaphase of the NBS cell line after irradiation with 1.0 Gy. **Figure S4** *hTERT* expression in diploid NBS-fibroblasts and SV40 transformed NBS cell lines. (DOCX 1.44 mb)

## Abbreviations

BrdU: Bromodeoxyuridine
CGH: Comparative genomic hybridization
DDR: DNA damage response
DSB: DNA double strand break
Gy: Gray
HRR: Homologous recombination repair
iPSCs: Induced pluripotent stem cells
MRN: MRE11/RAD50/NBN
NBS: Nijmegen breakage syndrome
NHEJ: Non-homologous end-joining
Q-FISH: Quantitative fluorescence in situ hybridization
TRF: Terminal restriction fragment
WCP: Whole chromosome painting
WGS: Whole genome sequencing
