# Supplementary material for: Evidence for a pre-malignant cell line in a skin biopsy from a patient with Nijmegen breakage syndrome
Source: Mol Cytogenet. 2018 Feb 7;11:17. doi: 10.1186/s13039-018-0364-6 (PMC5803995; doi:10.1186/s13039-018-0364-6)
Supplement: Additional file 1: Figure S1a,b. — Alignment of the cDNA spanning the breakpoints at chromosomes 6 and 13 and chromosomes 13 and 20. Figure S2. Metaphases of the NBS cell line after BrdU labelling for 36 and 72 h. Figure S3. Metaphase of the NBS cell line after irradiation with 1.0 Gy. Figure S4 hTERT expression in diploid NBS-fibroblasts and SV40 transformed NBS cell lines. (DOCX 1.44 mb) [file 13039_2018_364_MOESM1_ESM.docx]

**Additional Files**


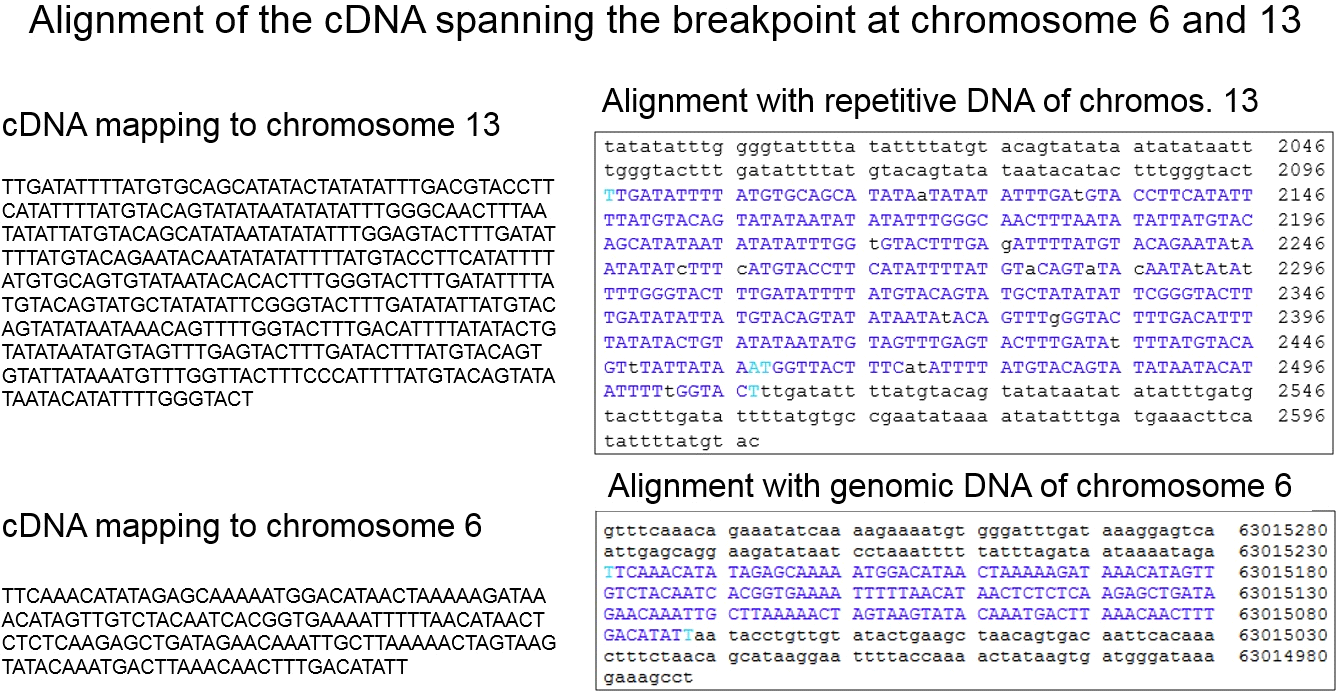


**Figure S1a**

Alignment of the cDNA spanning the breakpoint at chromosomes 6 and 13 with the genomic reference DNA of chromosome 6 (hg 19) and the human clone TRI-6, a satellite I repeat region, on chromosome 13.


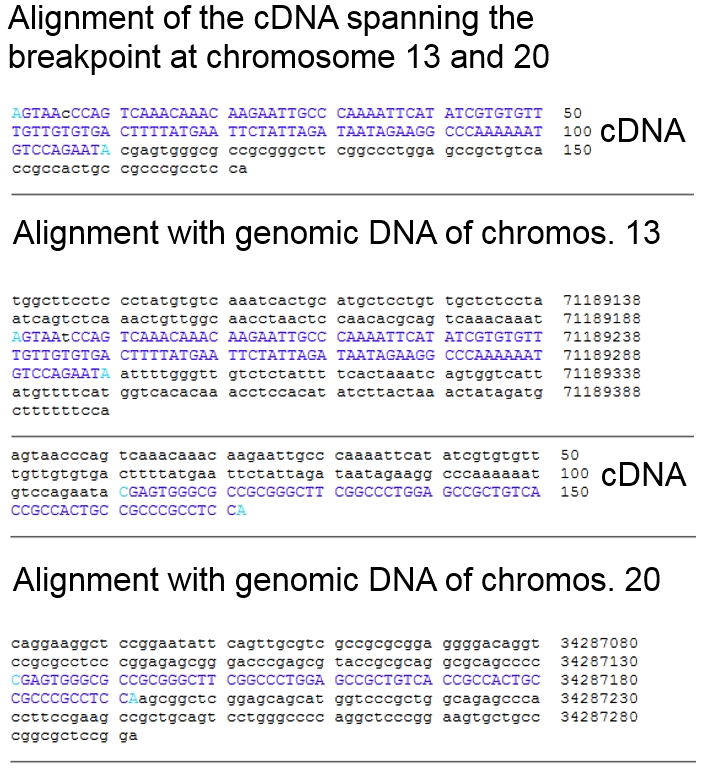


**Figure S1b**

Alignment of the cDNA spanning the breakpoint at chromosomes 13 and 20 with the genomic reference DNAs of both chromosomes.


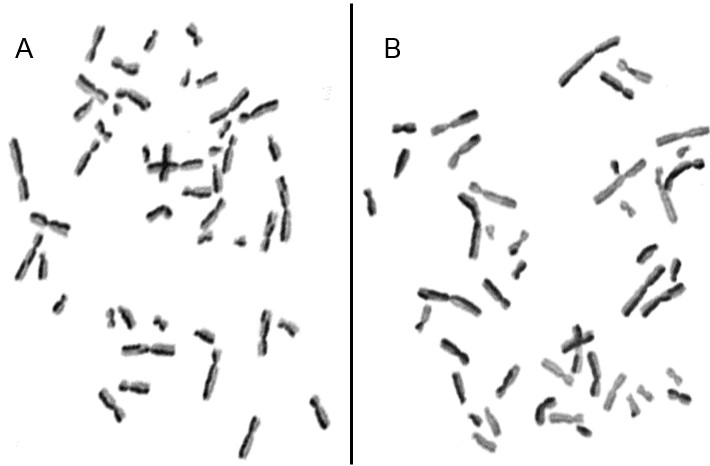


**Figure S2**

A. Metaphase of the NBS cell line 94P0496 after BrdU labelling for 36 hours. The chromosomes show the labeling pattern of a M2 metaphase with 6 sister chromatid exchanges.

B. M3 metaphase after 72 h BrdU labelling (Original from [16]).


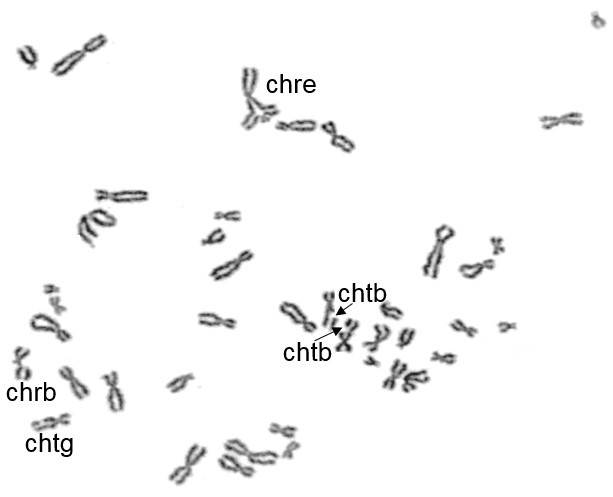


**Figure S3**

Metaphase of the NBS cell line 94P0496 after irradiation with 1.0 Gy 4 hours before harvesting (early G_2_ phase), showing one chromatid translocation (chre) two chromatid breaks (chtb), one isochromatid break (chrb), and one achromatic lesion (chtg) (Original from [16]).


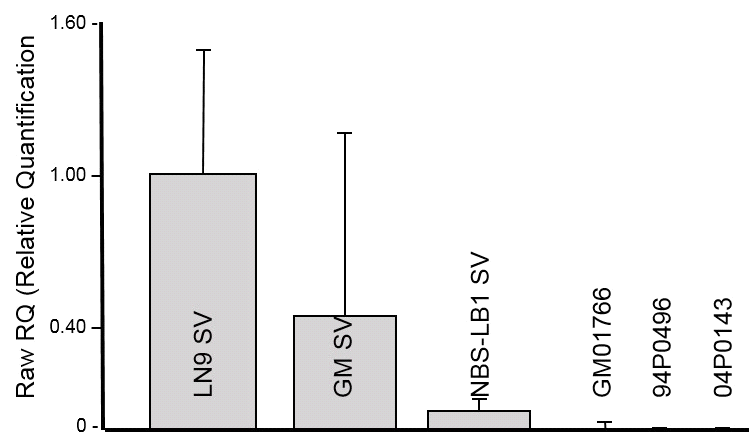


**Figure S4**

*hTERT* expression in NBS-fibroblast cell lines 94P0496, 04P0143 and GM01766 and two SV40 transformed NBS cell lines (GM SV, NBS-LB1SV) and a SV44 transformed control (LN9 SV). Original from [16].
